# Memory footprint: Predictors of flashbulb and event memories of the 2016 Euro Cup final

**DOI:** 10.3389/fpsyg.2023.1116747

**Published:** 2023-02-21

**Authors:** Andreia Ribeiro, Margarida Marques, Magda S. Roberto, Ana Raposo

**Affiliations:** Research Center for Psychological Science, Faculdade de Psicologia, Universidade de Lisboa, Lisbon, Portugal

**Keywords:** flashbulb memory, event memory, positive event, Euro Cup 2016, structural equation modeling

## Abstract

Two years after Portugal won the UEFA European Championship, we examined what the Portuguese remember of this momentous occasion. We investigated if flashbulb memories (FBMs) and event memories (EMs) were determined by distinct factors, and whether EM was a predictor of FBM. Participants responded to an online questionnaire about their FBM, EM and set of predictors. Structural equation modeling revealed that FBM and EM were associated with different pathways. Interest in football predicted importance which triggered emotional intensity which predicted personal rehearsal, a direct determinant of FBMs. On the other pathway, interest determined knowledge about football, the main predictor of EMs. Importantly, EM was a causal determinant of FBM which shows that the memory trace for the original event enhances memory for the reception context. The findings suggests that even though the two types of memories are determined by independent factors, they interact very closely.

## 1. Introduction

On July 10, 2016 Portugal’s national football team won for the first time the UEFA European Championship. The event was marked by Cristiano Ronaldo’s injury on the first half, which forced him off the match, and by Eder’s goal in extra-time, on an exciting twist, that led to the victory. By beating France, the host country, and given the importance of football in Portugal, the win was filled with emotion, much celebrated and talked about. Two years after, what memories do Portuguese people retain about this event? How vivid are their memories and how intense are their emotions? In the present study we examined this positive flashbulb memory.

Since the landmark work of Brown and Kulik (1977) flashbulb memory (FBM) has remained a central concept in cognitive and neuroscience research. It refers to the recollection of the personal circumstances in which one has learned of a significant public event (e.g., where you were when you first learned about the 9/11 attacks). Thus, the memories may vary greatly from one person to another even if related to the same occurrence (Brown and Kulik, 1977; Tinti et al., 2014). FBMs are typically vivid, long-lasting, and endowed with high confidence levels, as people often believe that the memories they recall are accurate (Brown and Kulik, 1977; Conway et al., 1994; Hirst and Phelps, 2016). Yet, research has revealed that FBMs are as susceptible to decay and distortions as other memories for everyday events (Talarico and Rubin, 2003; Hirst et al., 2009). Concomitantly, a noteworthy feature of FBMs is that confidence remains high even when the consistency of the details evoked declines over time, while confidence for other memories wanes with consistency. This divergence in confidence is thought to be associated with the vividness (recollections tend to be incredibly detailed even if inconsistent) and ease of retrieval accompanying FBMs (Talarico and Rubin, 2003; Hirst and Phelps, 2016).

Events that promote FBMs tend to be surprising, imbued with emotions, relevant for both the individual and the community, and frequently talked about (i.e., rehearsed) in private and publicly (Brown and Kulik, 1977; Conway et al., 1994; Tinti et al., 2014). Most FBM studies encompass emotionally negative events, presumably because it is easier to find significant public events with a negative, rather than a positive, connotation (Kraha and Boals, 2013). These studies have focused on the terrorist attacks of September the 11th in 2001 (e.g., Talarico and Rubin, 2003; Curci and Luminet, 2006; Hirst et al., 2009, 2015), the Paris attacks in 2015 (e.g., Gandolphe and El Haj, 2017), disasters like the Challenger explosion (Bohannon and Symons, 1992; Neisser and Harsch, 1992), and the death of public figures like Martin Luther King (Brown and Kulik, 1977), John F. Kennedy (Brown and Kulik, 1977), Olaf Palm (Christianson, 1989), Michael Jackson (Day and Ross, 2014), Pope John Paul II (Tinti et al., 2009; Lanciano et al., 2013), and Princess Diana (Hornstein et al., 2003). Nevertheless, positive events can also elicit FBMs (Tekcan, 2001; Stone and Jay, 2018). For example, people report vivid details of the circumstances in which they learned about key social and political events, considered to be positive for most participants, including the Danish liberation in World War II (Berntsen and Thomsen, 2005), the fall of the Berlin Wall (Bohn and Berntsen, 2007), the moon landing (Winograd and Killinger, 1983), the inauguration of Barack Obama as president (Koppel et al., 2013) and the death of Osama Bin Laden (Kraha et al., 2014; Demiray and Freund, 2015). Sporting events have also been considered to be a useful context for investigating positive FBMs, particularly for fans of winning teams (Kensinger and Schacter, 2006; Talarico and Moore, 2012; Tinti et al., 2014; Merck et al., 2020).

In addition to FBMs, significant public events are often associated with event memories (EMs), that is, memories for the factual details of the event (e.g., the number planes involved in the 9/11 attacks). Hence, FBM and EM differ with respect to their contents: FBM entails a first-person perspective and refers to the personal circumstances in which one learned about the event, whereas EM consists of factual information about the original event (Tinti et al., 2014). As such, contrary to FBMs that vary across individuals, accurate EMs should be identical for different people and, similarly to other types of memories, EMs decline over time (Bohannon and Symons, 1992; Hirst et al., 2015). To understand the cognitive processes that underlie FBM and EM, researchers have explored the factors that shape each type of memory. This is an important question as different determinants would indicate that both types of memory, even though related to the same event, are supported by independent mechanisms.

Structural equation modelling (SEM) is particularly useful to address this question. It allows comparing a theoretically-driven model with the empirical data, by assessing the extent to which the data fits the model. As such, SEM informs about which factors predict each type of memory and how the various factors relate to each other (Luminet, 2018). A number of models for the formation and maintenance of FBMs have been proposed and tested using SEM (Conway et al., 1994; Finkenauer et al., 1998; Er, 2003; Curci and Luminet, 2006; Luminet and Curci, 2009; Day and Ross, 2014; Tinti et al., 2014; for a review, see Luminet, 2018). They tend to agree on the set of variables that need to be considered, such as the emotional intensity of the event, the importance attributed to it, background knowledge, and how often memories are rehearsed through the media or in conversations. However, differences across models emerge concerning the relationship between these variables. According to Finkenauer et al. (1998), FBMs develop through two pathways. In the first pathway, the event is appraised in terms of *novelty* which leads to a reaction of *surprise* and *emotion* which in turn predict FBM. In the second pathway, the *importance* attributed to the event leads to intense *emotions* that trigger *rehearsal*. Background *knowledge* about the event also influences importance, emotions and rehearsal. Critically, according to this model, rehearsal strengthens EM which determines FBM.

Although research shows that Finkenauer et al. (1998) model provides the best fit with the data at least for negative events (see Luminet, 2018), we decided to test another model, proposed by Tinti et al. (2014), for three main reasons. First, this model has not yet been systematically validated and it was specifically elaborated to test a positive FBM which, similarly to our study, concerned the winning of the Italian football team during the 2006 World Cup. As noted earlier, the literature of FBM and positive events is rather limited, with some authors suggesting that the event’s valence may explain differences in results (Luminet, 2018). Second, sports events differ from other FBM events in that people prepare for the game, often watch the match unfold and discover the outcome at that time, whereas in most FBM events examined in the literature, events tend to be more unexpected and people hear about the outcome after the fact. As such, it is crucial to use a model that was specifically developed to explain memory for an event of a similar nature and valence (i.e., a sport’s event with a positive outcome), in order to be able to compare the findings. Third, Tinti’s model was the first to make the distinction between collective rehearsal (through the media) and individual rehearsal (social sharing and rumination). This is an important distinction that should be incorporated in the model, because the information that people recall from the game may be prone to corrections due to collective rehearsal.

Similarly to the influential model by Finkenauer et al. (1998), Tinti et al. (2014) have proposed a two-path model that distinguishes between FBM and EM determinants. A public event has to first capture people’s *interest* so that both FBMs and EMs are formed and maintained. From interest, two distinct paths to FBM and EM have been hypothesized. In the first, interest predicts the *importance* attributed to the event, and appraising an event as important and consequential evokes greater *emotional intensity*, which promotes greater *personal rehearsal*, i.e., thoughts and conversations about the circumstances in which the news were received. As the personal experience is rehearsed through thinking and talking, FBMs (as measured by vividness, number of details evoked and confidence) are strengthened. Indeed, the relationship between some of these factors and FBM has been demonstrated in earlier work. In the Brown and Kulik (1977) study, African Americans, when compared to White Americans, not only reported higher ratings of importance/consequentiality but also a greater proportion and more vivid FBMs related to the assassination of political leaders involved in civil rights. In another study, Conway et al. (1994), who targeted FBMs about Margaret Thatcher’s resignation, have shown that interest in politics and knowledge about Thatcher’s government predicted the importance attributed to the event and the affective response to the news, which in turn predicted FBM (Conway et al., 1994).

Regarding the second pathway, Tinti et al. (2014) have proposed that *interest* in the public event is often associated with greater *knowledge* about the event, which is certainly the case in sports events: the greater the interest, the more people know about the sport, the players, and the matches. Knowledge structures aid encoding and integration of new information, hence improving EM (i.e., greater accuracy and certainty). By comparing memory for the death of former French President, François Mitterrand, in French and Belgian participants, Curci et al. (2001) showed that the first group had more knowledge about Mitterrand and his politics, independently of personal interest in French politics, and displayed more FBMs. Yet, Tinti et al. (2014) argued that knowledge did not impact FBM, but rather it fostered assimilation and organization of information which in turn improved EM. Tinti’s model also accounts for an alternative path to EM enhancement. *Interest* leads to an appraisal of the event’s *importance* which triggers *media rehearsal*, that is, searching and being exposed to factual aspects of the event (e.g., who scored a goal). This repeated consultation of information about the event across different media enhances EM. Indeed, media rehearsal can modify memories and correct incongruencies leading to more accurate EMs (Hirst et al., 2009, 2015; Tinti et al., 2014; Hirst and Phelps, 2016). Based on these findings and perspectives, our first goal was to test the model proposed by Tinti et al. (2014), by investigating the extent to which FBM and EM develop through distinct pathways.

Another critical question concerns the role of EM upon FBM. In Tinti et al. (2014), EM was not a causal determinant of FBM. According to the authors, this lack of relationship explains why FBMs are often vivid and yet error-prone, whereas EMs tend to be corrected (notably, through media exposure). Nevertheless, this result stands in stark contrast with other existing work that have reported a significant positive association between EM and FBM (e.g., Finkenauer et al., 1998; Er, 2003; Tinti et al., 2009). In fact, EM has been pointed out as one of the most consistently significant predictors of FBM (see Luminet, 2018 for a review). Hence, our second goal was to determine the role of EM upon FBM and in this way help to identify the direct and indirect paths to FBM.

Although Tinti’s model was specifically intended to assess FBMs for positive events, it has some methodological limitations that we took into consideration and attempted to overcome in our study. First, they measured FBM as a composite score computed based on the number of details evoked, degree of vividness, and degree of certainty. Although these indexes are considered important dimensions of FBM, the data show that they are independent of each other and thus should not be analyzed together as a single measure of FBM (see Luminet, 2018, for an extensive discussion). Second, the number of details evoked was calculated based on the total number of details included in the participants’ accounts, which has been considered a simplistic measurement procedure to assess FBM (Neisser and Harsch, 1992). Alternatively, a Weighted Attribute Score (WAS) has been proposed where different weights are assigned depending on the type of FBM detail evoked. This score assumes that the information recalled is not all equally important, with some attributes considered to be “major,” i.e., canonical features essential to identifying the reception context (e.g., location, ongoing activity) while others are taken as “minor” or peripheral (e.g., other people present). The score gives more weight to the canonical features than the peripheral attributes of FBM, and it represents a measure of overall precision and detail of FBM. The WAS system has been extensively used (Pezdek, 2003; Smith et al., 2003; Tekcan et al., 2003; Shapiro, 2006; Kvavilashvili et al., 2009, 2010; Merck et al., 2020), and is seen as an advancement toward a measurement model of FBM (Curci, 2018). Hence, we employed the WAS system for a more fine-tuned measurement of FBM.

In sum, the present study investigated the memories of Portuguese citizens for the 2016 European Football Championship victory, 2 years after the event occurred. This study adds to the extant literature on FBMs for sporting events, making two main contributions: (1) To test the two-path model proposed by Tinti et al. (2014) in order to assess the extent to which FBM and EM are determined by different predictors. Importantly, we will account for some of the limitations of the model and will employ an improved procedure to assess FBM, which will help overcome the methodological criticisms the model has faced. (2) To determine the extent to which EM predicts FBM in the context of a positive event. Given the inconsistent findings in the literature, further examination of this link is warranted. Establishing if the memory trace for the original event enhances memory for the reception context is a critical endeavor to elucidate the relationship between the two types of memory.

We expect that FBM and EM have different determinants, with the former being influenced by importance, emotional intensity, and personal rehearsal, and the latter by knowledge and media rehearsal. We further hypothesize that EM is a causal determinant of FBM, since during learning and rehearsal of the original event, all information associated with it, including the reception context and the factual details, should be activated strengthening the link between the two types of memory (Finkenauer et al., 1998).

## 2. Materials and methods

### 2.1. Participants

A total of 245 participants filled in a questionnaire, 23 of whom were excluded from the data analysis. The majority (*n* = 20) were excluded for failing to complete the survey and the remainder (*n* = 3) for having a nationality other than Portuguese. Of the 222 participants included in the analysis, all were Portuguese citizens, recruited both at Universidade de Lisboa and online using the snowball sampling method. Their ages ranged from 18 to 85 years old (*M* = 28.58 years, *SD* = 14.61), and 63.50% were females. 82.40% of the participants lived in the Lisbon metropolitan area. 76.60% were fans of a football club (37.80% were supporters of Sport Lisboa e Benfica and 30.60% were fans of Sporting Clube de Portugal, the biggest teams in the Lisbon area). Participants were tested between April and November of 2018, about 2 years after the 2016 UEFA European Championship. All reported having watched the entire game of the final, while 53.60% stated having watched all matches played by Portugal. Students from Universidade de Lisboa received a course credit as compensation for their participation. All participants were informed that the questionnaire was anonymous, and the data would be used for research purposes only. The study was approved by the Ethics Committee of Faculdade de Psicologia of Universidade de Lisboa.

### 2.2. Procedure

Participants read the informed consent and were briefed about the confidentiality, the main goal of the study and the criteria for participation (i.e., being a Portuguese citizen, being 18 years old or more, and having watched the entire game of the final). Then, they proceeded to the different sections of the questionnaire (described below). Survey completion was online, using Qualtrics Software (Qualtrics, Prove, UT), and took on average 23 min (*SD* = 21.94).

### 2.3. Measures and coding

The questionnaire assessed FBMs, EMs and six possible determinants of these memories. To decide which determinants to include, we relied on previous studies (e.g., Brown and Kulik, 1977; Conway et al., 1994; Tinti et al., 2014).

#### 2.3.1. Flashbulb memory: Detail, confidence, and vividness

Participants were asked 10 open questions about their personal memories for the final game of the 2016 UEFA European Championship. Six questions focused on the canonical characteristics previously described in the literature (Brown and Kulik, 1977; Kızılöz and Tekcan, 2013): where they were, with whom they were, how they felt when they heard about the victory, how other people around them reacted, what they did immediately after the game, what they did immediately before the game.^1^ Another four questions entailed peripheral information about the context where the participants experienced the victory: what they ate and drank, what they were wearing, with how many people they were (see Supplementary Table S1). For each answer that was provided, participants indicated how confident they were in their responses using a 7-point scale ranging from 1 (not at all confident) to 7 (very confident). To measure FBM vividness, participants indicated how vivid was their image of the moment in which they learned that Portugal had won the European Championship, using a 7-point scale where 1 = not at all vivid and 7 = very vivid. The mean confidence rating was used as an indicator of confidence in the FBM evoked (FBM_Confidence) while the vividness rating was taken as an index of vividness of the FBM (FBM_Vividness).

To score the FBM details, we employed the WAS procedure, proposed by Neisser and Harsch (1992), in which different weights are assigned to different details evoked. Each response was scored 2 if the participant responded by providing details (e.g., “in the holiday home of my best friend”), 0 if she/he did not respond, and 1 for intermediate cases (e.g., “I was outside”). The WAS is the sum of the scores on the six major attributes (maximum of 12 points), plus two bonus points awarded if participants score 6 or more (of 8 possible) on the minor details. In this way, WAS clearly differentiates between major (canonical) and minor (peripheral) details, by attributing a maximum of 12 points for the recall of critical information and a maximum of 2 points for recalling less critical information. The score thus ranges from 0 to 14 and it was used as a measure of the details remembered (FBM_Detail). Two independent judges (co-authors of the paper) coded the answers. To assess reliability, we used the R package irr (Gamer et al., 2019). The intraclass correlation coefficient (ICC) was computed with a two-way random ANOVA model to measure absolute agreement based on ratings of the two coders [*F*(221, 6.37) = 13.5, *p* = 0.001]. The ICC was 0.78 (95% *CI* [0.36, 0.90]), suggesting good reliability (Koo and Li, 2016). We also computed the mean of Pearson’s correlations (*r*) between raters as an index of reliability with r to Fisher-z transformation before averaging. The index revealed a strong inter-rater correlation (*r* = 0.88, *z* = 13, *p* < 0.001). Finally, the internal consistency for the set of ratings was checked using coefficient alpha (α = 0.93), which was high (Nunnally and Bernstein, 1994).

#### 2.3.2. Event memory: Accuracy and confidence

Ten open questions were used to measure participants’ ability to recall factual information about the game (e.g., How many goals were scored? In what city was the game?; see Supplementary Table S1 for a complete list of the questions). Responses were scored 1 if correct and 0 if incorrect. As frequently done in SEM analyses involving scales with items scored as correct or incorrect, items were parceled, that is, aggregated into two “parcels” which were used as indicators of the latent construct. This procedure enhances model parsimony and can improve the quality of indicators and model fit (Landis et al., 2000; Bandalos and Finney, 2001). For that, we constructed two equivalent event-memory sub-scales by considering the proportion of correct odd items and even items separately (as in Tinti et al., 2014). Accuracy of EM was thus measured by the proportion of correct responses to the five odd questions (EM_Accuracy1) and the proportion of correct responses to the five even questions (EM_Accuracy2). After each response, participants indicated how confident they were in their answer using a 7-point scale. To balance the number of accuracy and confidence measures (providing two measures of each), we calculated the mean confidence for each participant separately for the odd items (EM_Confidence1) and the even items (EM_Confidence2).

#### 2.3.3. Determinants of flashbulb memory and event memory

A set of questions assessed the potential determinants of FBM and EM.

##### 2.3.3.1. Interest

Participants were asked to indicate how strongly they supported their football team (Support_Team) and how frequently they watched football games (Follow_Football), using 7-point scales.

##### 2.3.3.2. Importance

Participants indicated how important the victory was for them (Personal_Importance), for family members (Family_Importance), to Portugal (National_Importance) and to the international community (International_Importance) using a 7-point scale, in which 1 = not at all important and 7 = very important.

##### 2.3.3.3. Emotion

Participants were asked to think about the moment in which they learned about Portugal’s victory and to rate the intensity of their emotional reaction where 1 = not at all intense and 7 = very intense (Emotional_Intensity). Besides, they rated 10 discrete emotions (5 positive and 5 negative) using the same 7-point scale. The mean rating for pride, relief, satisfaction, happiness and fulfilment was used as a measure of the intensity of positive emotion (Pos_Emotions), whereas the mean rating for sadness, anger, fear, regret and disgust was considered an indicator of the intensity of negative emotion (Neg_Emotions).

##### 2.3.3.4. Personal rehearsal

Participants rated how frequently they thought and talked about the victory within the first 24 h after the game (Rehearsal_24 h), and in the past 6 months (Rehearsal_6M). Answers were given in a 7-point scale ranging from 1 (very rarely) to 7 (very frequently).

##### 2.3.3.5. Media rehearsal

Participants indicated how frequently they followed the news about the victory through media (television, social networks, newspapers, and radio) within the first 24 h after the game (Media_24h), and in the past 6 months (Media_6M), using a 7-point scale.

##### 2.3.3.6. Surprise

Participants rated how surprised they felt about Portugal’s victory, using a 7-point scale, ranging from 1 (not at all surprised) to 7 (very surprised).

##### 2.3.3.7. Knowledge about football

Participant’s general knowledge about football was evaluated through 10 open questions (e.g., How frequent is the European Championship? How many substitutes are allowed in a game?; Supplementary Table S1). Responses were scored 1 if correct and 0 if incorrect. The same procedure of item parceling used for event memory was implemented. Hence, knowledge about football was measured as the proportion of correct responses to the five odd questions (Know_Accuracy1) and to the five even questions (Know_Accuracy2).

### 2.4. Data analysis

**Figure 1:**
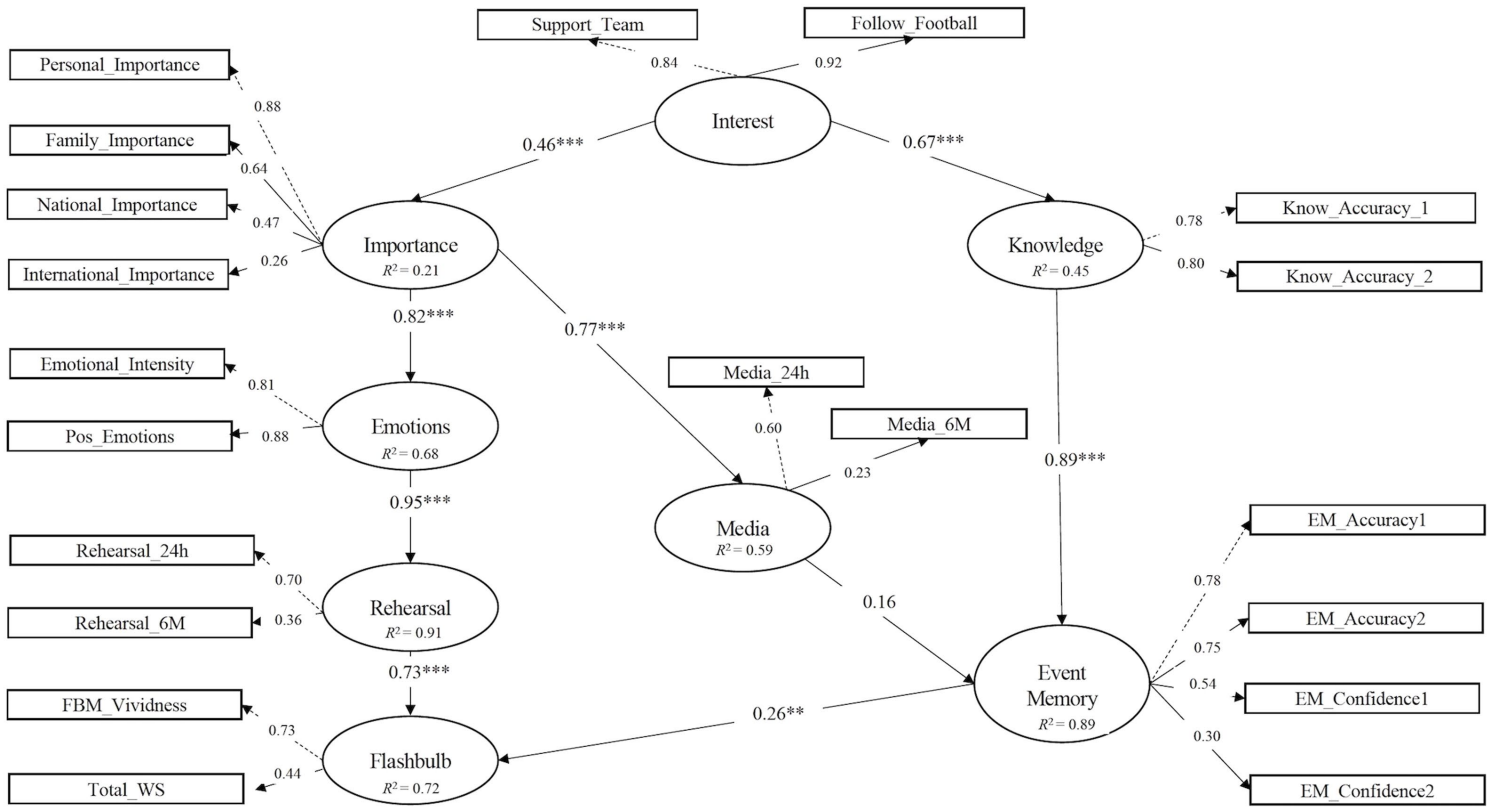
Standardized factor loadings and regression coefficients of the empirical model of flashbulb memory and event memory. **p < 0.01; ***p < 0.001.

Data analysis was conducted in two steps. We first analyzed the descriptive statistics of FBM, EM and the predictors measured (Table 1). Then, SEM was used to test the hypothesized model (Figure 1) comprising a structural part (relationships between latent variables) and a measurement part (relationships between latent variables and their indicators). Regression coefficients as well as factor loadings were estimated, respectively. Anticipating strong correlation between indicators defining the latent variables personal rehearsal and media rehearsal (as they denote two types of rehearsal), covariances between error terms were included. Distributional assumptions were checked using graphical representations (quantile-quantile plots) with deviations from the normal distribution being considered in the presence of extreme values (|z| > 3; Kline, 2011). In addition to the significance (*p <* 0.05) of the hypothesized relationships and the model chi-square (χ^2^), adjustment was assessed using the following indices: the Tucker Lewis fit index (TLI; Tucker and Lewis, 1973), the comparative fit index (CFI; Bentler, 1990), the root mean square error of approximation (RMSEA; Browne and Cudeck, 1992) with 90% confidence interval (CI), and the standardized root mean square residual (SRMR; Jöreskog and Sörbom, 1988). Acceptable model fit to the data occurred when CFI and TLI values were equal or greater than 0.90, with RMSEA and SRMR values being less than 0.08 (Hu and Bentler, 1998). Modification indices suggesting model alterations were analyzed and included in the model only if theoretically justifiable. To compare competing models, we used the Bayesian information criteria (BIC; Schwarz, 1978) to account for model complexity with models with lower BIC values suggesting better fit. R-square (*R*^2^) values for the dependent latent variables in the models were also computed. SEM was performed using the lavaan package (Rosseel, 2012) designed for R environment (R Core Team, 2021).

**Table 1:** Descriptive statistics of the variables and indicators.

**Variable**	**Indicator**	***M***	***SD***
Flashbulb memory	FBM_Detail (0–14)	12.51	1.80
	FBM_Confidence (1–7) *	6.47	0.61
	FBM_Vividness (1–7)	5.69	1.31
Event memory	EM_Accuracy1	0.54	0.26
	EM_Accuracy2	0.23	0.24
	EM_Confidence1 (1–7)	5.02	1.51
	EM_Confidence2 (1–7)	4.60	1.77
Interest	Support_Team (1–7)	5.14	1.94
	Follow_Football (1–7)	3.87	2.22
Importance	Personal_Importance (1–7)	4.82	1.80
	Family_Importance (1–7)	4.48	1.69
	Nation_Importance (1–7)	6.59	0.81
	International_Importance (1–7)	5.24	1.79
Emotion	Emotional_Intensity (1–7)	6.16	1.08
	Pos_Emotions (1–7)	5.94	0.96
	Neg_Emotions (1–7) *	1.18	0.44
Rehearsal	Reharsal_24h (1–7)	5.83	1.44
	Rehearsal_6M (1–7)	2.69	1.48
Media	Media_24h (1–7)	5.39	1.71
	Media_6M (1–7)	2.75	1.52
Surprise *	Surprise (1–7) *	5.24	1.38
Knowledge	Know_Accuracy1	0.38	0.25
	Know_Accuracy2	0.44	0.30

Variables and indicators marked with * were not included in the best fitting SEM.

## 3. Results

### 3.1. Descriptive statistics

The means and standard deviations for the items are reported in Table 1. With respect to FBM, participants responded to 88% (*SD* = 13%) of the questions, indicating that their personal memories were quite detailed. The mean WAS was 12.51 (*SD* = 1.80). Almost all participants were able to report where they were (99.50%), with whom they were (99.10%), how they felt (97.70%), and how others around them reacted (99.10%). Indeed, an impressive 39.6% of the participants were able to respond to all FBM questions asked, denoting the recall of canonical and peripheral details. Participants reported vivid memories of the moment in which they learned about Portugal’s victory (*M* = 5.69; *SD* = 1.31) and high confidence in the evoked memories (*M* = 6.47, *SD* = 0.60), even 2 years after the event. For EM, participants provided, on average, correct answers to 39% (*SD* = 22%) of the questions, with an overall moderately high confidence rating (*M* = 4.81, *SD* = 1.64). One participant correctly recalled all the information prompted about the game. Gender had a significant impact on EM accuracy, *t*(220) = −5.15, *p* < 0.001), with better performance for male (*M* = 0.49) than female participants (*M* = 0.34). Yet, gender did not affect the proportion of FBM reported, *t*(220) = −1.21, *p* = 0.23 (male: *M* = 0.89; female: *M* = 0.87). Participants’ age was not significantly correlated with either FBM (*r* = − 0.09, *p* = 0.17) or EM (*r* = −0.05, *p* = 0.42).

With respect to interest, participants judged their support for their team as high (*M* = 5.14, *SD* = 1.94), whereas how frequently they followed football games was evaluated as being lower (*M* = 3.87, *SD* = 2.22). Participants evaluated the victory as important, particularly for the nation (*M* = 6.59, *SD* = 0.81). They reported having had a strong emotional reaction when they learned of Portugal’s victory (*M* = 6.16, *SD* = 1.08). Specifically, they reported having felt an intense positive emotion (*M* = 5.94, *SD* = 0.96) whereas the negative emotion was of low intensity (*M* = 1.18, *SD* = 0.44). Participants’ personal rehearsal about the circumstances in which they learned about the victory was highly frequent in the 24 h after the game (*M* = 5.83, *SD* = 1.44), but low in the last 6 months (*M* = 2.69, *SD* = 1.48). Rehearsal *via* the media showed a similar pattern (*M* = 5.39, *SD* = 1.71 in the first 24 h; *M* = 2.75, *SD* = 1.52 in the last 6 months). The mean rating of how surprised they were about Portugal’s victory was 5.24 (*SD* = 1.38). Finally, the average proportion of correct responses to general knowledge questions was 41% (*SD* = 27%), with the relatively large standard deviation indicating that the amount of knowledge about football varied considerably across participants.

### 3.2. Structural equation models

Visual inspection of quantile-quantile plots suggested deviations from the normal distribution. Under non-normality, the Satorra-Bentler scaled chi-square (S-B χ2) was computed using the robust maximum likelihood estimator (MLR). We evaluated the empirical model proposed by Tinti et al. (2014), which hypothesizes that FBM and EM have different determinants, with the former being influenced by importance, emotional intensity, and personal rehearsal, and the latter by knowledge and media. Also, the model allows testing if EM is a causal determinant of FBM. Some improvements were incorporated relative to the original model. First, as explained earlier, our score of FBM details contemplated the WAS procedure (rather than the simple sum of details). Second, the variable flashbulb memory included two indicators, details evoked (FBM_Details) and degree of vividness (FBM_Vividness), which were moderately correlated (*r* = 0.38, *p* < 0.001). We excluded the degree of confidence (FBM_Confidence) as it showed a weak correlation with details (*r* = 0.13, *p* = 0.045), suggesting an independence between these items which therefore should not be analyzed together (Luminet, 2018). Third, the variable surprise was not included in our model as it showed no significant effects in Tinti et al. (2014) study, possibly because the football match is a predictable event (Curci and Luminet, 2009), and because we only collected a single item evaluating surprise (making it unsuitable for inclusion in the SEM).

The model showed a poor fit to the data (S-B χ2 (178) = 442.30, *p* < 0.001, CFI = 0.84, TLI = 0.81, RMSEA = 0.09, 90% *CI* RMSEA = [0.08, 0.10], SRMR = 0.09, BIC = 10039.44). To improve model fit, we inspected modification indices, which suggested the inclusion of two within-factor error covariances: one depicting the association between EM_Confidence1 and EM_Confidence2 (which define the latent variable event memory), and one other modeling the correlation between Personal_Importance and Family_Importance (which define the latent variable importance). Also, the indicator negative emotions (Neg_Emotions) did not load significantly (*p = 0*.139) on the latent variable emotions and was removed. The fit indices of the respecified model improved, suggesting a reasonable model fit to the data (S-B χ2 (156) = 257.81, *p* < 0.001, CFI = 0.94, TLI = 0.92, RMSEA = 0.06, 90% *CI* RMSEA = [0.05, 0.07], SRMR = 0.08, BIC = 9744.87). Regarding the model measurement part, all factor loadings were significant (*p* < 0.05). As for the model structural part, all paths were significant, including the path linking EM and FBM (*p* = 0.008). The only path that was statistically not significant was the one linking media and EM (*p* = 0.073). Overall, this model explained 72% of the variance for FBM and 89% of the variance for EM. The standardized factor loadings and regression coefficients for this model are depicted in Figure 1.

## 4. Discussion

We investigated the memories of Portuguese citizens for the victory of the national football team in the 2016 European Championship. Specifically, we examined which predictors determined FBM and EM, and assessed the role that EM plays on FBM. For that, we tested the model of Tinti et al. (2014), which was specifically developed for a positive sporting event like ours, while including some methodological adaptations which improved the way FBM was operationalized.

Consistent with earlier work on FBMs, we found that people provided several details about the circumstances in which they learned about the victory, evoking on average 88% of the probed information. The details recalled included the canonical categories defined by Brown and Kulik (1977), such as where they were, with whom they were, and how they felt, but also some trivial details like the colour of their clothes and what they drank and ate during the game. Overall, these memories were rated as very vivid and participants were quite confident in their accuracy, as it has been reported for other momentous occasions (e.g., Rubin and Kozin, 1984; Bohannon, 1988; Talarico and Rubin, 2003; Gandolphe and El Haj, 2017). These findings confirm that this positive event possess flashbulb characteristics and that Portuguese citizens were able to report their personal memories of that moment 2 years after the game. In contrast to FBMs, EMs of the match were reported to a lesser extent and had a low accuracy rate (i.e., 39%). A similar result was found in previous studies (Bohannon and Symons, 1992; Smith et al., 2003; Tekcan et al., 2003), including in Tinti et al. (2014) where the mean correct recall for the factual details of the 2006 World Cup final was 3.1 in a scale ranging from 0 to 6. Moreover, whereas 39.6% of the participants (88 out of 222) were able to evoke all FBM details prompted in the questionnaire, only one participant responded correctly to all EM questions. The mean confidence for correctly retrieved EMs was lower than for the FBMs evoked. Together, these findings indicate that participants were able to provide more details for the personal circumstances in which they learned about the event than for the event itself and did so more confidently.

SEM revealed that FBMs and EMs were shaped by distinct factors. In line with our hypothesis, interest in football predicted the importance attributed to the game, which triggered emotional intensity which in turn predicted personal rehearsal, a direct determinant of FBMs. Importance and emotional intensity have for long been considered key determinants of FBMs (Brown and Kulik, 1977; Neisser and Harsch, 1992; Er, 2003; Talarico and Rubin, 2003). As for rehearsal, speaking with others and thinking about the event are moments of memory retrieval. It is well known that retrieval practice modifies memories, by strengthening or altering old memories, creating new ones or inducing forgetting (McDermott, 2006; Coman et al., 2009). This memory reconstruction that occurs during rehearsal may thus explain why, independently of accuracy, FBMs are associated with high confidence and vividness.

Turning to EMs, our finding corroborated the hypothesis that prior knowledge was the primary determinant of EM. Several studies have shown that semantic knowledge (i.e., schemas) benefit learning of new episodic information by providing a scaffold into which new related information can be anchored (Bartlett, 1932; Kan et al., 2009; Van Kesteren et al., 2012). For example, knowing who the players of different teams are presumably helps remembering who disputed the ball in a specific moment of the match. Yet, most work on how prior knowledge supports memory has been conducted within the episodic memory literature, particularly in laboratory-based studies. Evidence for the role of knowledge in FBM is scarcer (Conway et al., 1994; Curci et al., 2001; Tinti et al., 2014) and therefore should be considered more systematically in future research.

Contrary to our hypothesis, in the present study, frequency of media rehearsal did not arise as a significant predictor of EM. We should note that the loading on the media latent variable was very low for the media exposure in the last 6 months (0.23 as illustrated in Figure 1), suggesting that this item may not be adequate to measure the latent variable. Relatedly, while media coverage was intense shortly after the match, it faded with time. As Hirst et al.’s (2009) contrast between EM for 9/11 and the Challenger disaster suggests, accurate EM may depend on continuous coverage. As such, it is likely that the limited media coverage of the match over time may explain the lack of a significant effect between media exposure and EM and the relatively low performance of participants in EM questions (*M* = 39%) 2 years after the match. Moreover, Hirst et al. (2015) have shown that 10 years after 9/11, EM accuracy was only mediated by the level of media attention of the past 7 years. The amount of media exposure shortly after the event (1 week, 1 year, and 2 years after the event) did not correlate with EM 10 years later. These results suggest that long-term EM depends on recent (but not initial) media exposure and that continuous coverage of the news may be a critical factor for accurate EM.

Importantly, as predicted, EM was a significant determinant of FBM (Finkenauer et al., 1998; Er, 2003; Tinti et al., 2009; Hirst and Phelps, 2016; Luminet, 2018). It has been proposed that when learning about a new event, all information that constitutes that event, including the reception context, sensory and emotional information, and factual elements, are encoded in memory (Tulving and Kroll, 1995). As such, the factual information about the event and the context of its reception interplay very closely, constituting different elements of the entire experience. Hence, it is not surprising that the more information people have about the match, the more detailed, and vivid are their FBMs. Of note, previous studies that reported an association between EM and FBM targeted negative events. Here, we extend this finding to a positive event, and demonstrate that the lack of a significant relationship in Tinti et al. (2014) cannot be explained by the event’s valence. In his review, Luminet (2018) points out that the way FBM was operationalized by Tinti et al. (2014) may underlie such result. Indeed, by overcoming such limitations and using more reliable methods (i.e., combining only measures that correlate with each other and using the WAS procedure), the significant association between EM and FBM emerged.

Some important limitations of the current study should be mentioned. The assessment of memory and its determinants was done in a single shot, 2 years after the game. Although some studies have been conducted several months or years after the original event occurred and with a single time measurement (e.g., Finkenauer et al., 1998; Kopietz and Echterhoff, 2014; Tinti et al., 2014), it is generally agreed that models should include a consistency measure with the first measurement occurring immediately after the event (see Luminet, 2018 for a discussion). It is possible that 2 years after the game, memories have been modified and reconstructed through personal and media rehearsal. Hence, future studies should explore whether the current model explains FBM and EM when testing occurs immediately after the event and include a consistency measure of FBM and EM, in a test–retest paradigm. It is also noteworthy that surprise was not included in our model as a predictor, since sporting events are predictable, matches are scheduled and people often prepare for them. This is in contrast with other FBM events that tend to be unexpected and where surprise has been shown to be a critical predictor of FBMs (Finkenauer et al., 1998) and EMs (Congleton and Berntsen, 2022). As such, the lack of an association between surprise and memory is likely to be restricted to sports. To make these events more akin to other FBM events, it would be interesting, in future studies, to include participants who have not watched the match and only learned about it after the event unfold. In this context, surprise may emerge as a key predictor. Lastly, prior work has shown that some individuals with the so-called “highly-superior autobiographical memory” (HSAM) have extremely accurate memory for public events (LePort et al., 2016; Levine et al., 2021; Santangelo et al., 2021). Although our study was not designed to investigate this issue, we found that one participant was able to remember correctly all information prompted about the match. An interesting goal for future research includes finer-grained analyses of individual differences in both FBM and EM with a focus on HSAM individuals. Some open questions that are pertinent to address concern whether HSAM individuals excel in both types of memory tasks, the extent to which their memories are more consistent over time than memory of control participants, and how different factors (such as, emotional intensity, rehearsal) influence their long-term memories of public events.

In summary, our data provide a wide-angle view on the impact of different predictors on FBM and EM for a positive event. Corroborating the model proposed by Tinti et al. (2014), the two types of memory were shaped by distinct factors, suggesting that they reflect different memory processes. While FBM was predicted by importance, emotional intensity and personal rehearsal, EM was determined by prior knowledge. Importantly, EM was a significant predictor of FBM, suggesting that even though the two types of memories are determined by independent factors, they interact very closely.

## Data availability statement

The datasets presented in this study can be found in online repositories. The names of the repository/repositories and accession number(s) can be found at: https://osf.io/kanv8/?view_only=a23959f285694f2c9fe2ba9888150bdb.

## Ethics statement

The studies involving human participants were reviewed and approved by Comissão de Ética e de Deontologia, Faculdade de Psicologia, Universidade de Lisboa. The patients/participants provided their written informed consent to participate in this study.

## Author contributions

ARi developed the research design, programmed and supervised data collection, conducted the data analysis, and was involved in the discussion of the theoretical implications of this research. MM and MR conducted the data analysis and contributed to the discussion of the theoretical implications of this research. ARa conceived the research question, developed the research design, conducted the data analysis, and contributed to the discussion of the theoretical implications of this research. All authors contributed to the writing of the manuscript, revised the manuscript, and approved the final version.

## Funding

This work was supported by FCT – Fundação para a Ciência e a Tecnologia (Foundation for Science and Technology of Portugal) through a grant to the Research Center for Psychological Science of the Faculty of Psychology, University of Lisbon (UIDB/04527/2020; UIDP/04527/2020), and through a Ph.D. Studentship (2022.14147.BD) to ARi.

## Conflict of interest

The authors declare that the research was conducted in the absence of any commercial or financial relationships that could be construed as a potential conflict of interest.

## Publisher’s note

All claims expressed in this article are solely those of the authors and do not necessarily represent those of their affiliated organizations, or those of the publisher, the editors and the reviewers. Any product that may be evaluated in this article, or claim that may be made by its manufacturer, is not guaranteed or endorsed by the publisher.

## Supplementary material

The Supplementary material for this article can be found online at: https://www.frontiersin.org/articles/10.3389/fpsyg.2023.1116747/full#supplementary-material

## Footnotes

^1^Taking into account the nature of the event at hand, a football game with a scheduled time, the canonical category of ongoing activity was translated into the activity before the game. This decision is in consonance with studies that used the question about the prior activity as a way to address the activity that was interrupted by the event (e.g., Davidson and Glisky, 2002; Talarico et al., 2019).
