# Supplementary material for: Memory footprint: Predictors of flashbulb and event memories of the 2016 Euro Cup final
Source: Front Psychol. 2023 Feb 21;14:1116747. doi: 10.3389/fpsyg.2023.1116747 (PMC9990819; doi:10.3389/fpsyg.2023.1116747)
Supplement: Supplementary file 1 [file Table_1.pdf]

Supplementary Material

Table S1

*Questions Used to Evaluate Flashbulb Memory, Event Memory and General Knowledge About Football*

| |
|---|
| **Flashbulb Memory Questions** |
| 1. Where did you watch the match? * |
| 2. With whom were you with? * |
| 3. How many people were with you? |
| 4. How did you feel when you found out that Portugal won the Euro Cup? * |
| 5. How did other around you reacted? * |
| 6. What did you do after the match? * |
| 7. What did you do immediately before the match? * |
| 8. Did you eat during the match? If so, what did you eat? |
| 9. Did you drink during the match? If so, what did you drink? |
| 10. Which clothes were you wearing during the match? |
| **Event Memory Questions** |
| 1. How many goals were scored during the final? |
| 2. In the end of the 90 minutes, what was the score? |
| 3. In what city was the game? |
| 4. What is the referee’s nationality? |
| 5. What were the colours of the French team? |
| 6. Which pattern was in Ricardo Quaresma’s hairstyle? |
| 7. What was the colour of the gloves of the Portuguese goalkeeper? |
| 8. Which player did Éder replace when he got off the bench? |
| 9. Which insects flew around in the stadium? |
| 10. How many yellow cards were shown to the Portuguese team? |
| **General Knowledge Questions** |
| 1. How frequent is the European Championship? |
| 2. How many players of each team can go to the Euro Cup? |
| 3. Which countries won more Euro Cup tournaments? |
| 4. In which year was the Euro Cup played in Portugal? |
| 5. Which team won the last Euro Cup (in 2012)? |
| 6. How many substitutes are allowed in a game? |
| 7. How many teams play in the Portuguese football league? |
| 8. How long can the goalkeeper hold the ball? |
| 9. What many times did José Mourinho win the Champions? |
| 10. Which Portuguese players have won the Ballon d’Or? |

*Note*: Questions marked with * were considered to be canonical, representing major attributes of flashbulb memories.
